# Overcoming Rooming-In Barriers: A Survey on Mothers' Perspectives

**DOI:** 10.3389/fped.2020.00053

**Published:** 2020-02-21

**Authors:** Alessandra Consales, Beatrice Letizia Crippa, Jacopo Cerasani, Daniela Morniroli, Martina Damonte, Maria Enrica Bettinelli, Dario Consonni, Lorenzo Colombo, Lidia Zanotta, Elena Bezze, Patrizio Sannino, Fabio Mosca, Laura Plevani, Maria Lorella Giannì

**Affiliations:** ^1^Fondazione IRCCS Ca' Granda Ospedale Maggiore Policlinico, NICU, Milan, Italy; ^2^Department of Clinical Sciences and Community Health, University of Milan, Milan, Italy; ^3^Fondazione IRCCS Ca' Granda Ospedale Maggiore Policlinico, Epidemiology Unit, Milan, Italy; ^4^Fondazione IRCCS Ca' Granda Ospedale Maggiore Policlinico, Direzione Professioni Sanitarie, Milan, Italy

**Keywords:** rooming-in, maternal knowledge, barriers, facilitators, breastfeeding

## Abstract

**Background:** The importance of rooming-in in promoting breastfeeding initiation and continuation within the 10 Steps for Successful Breastfeeding is widely acknowledged. However, adherence to this practice by healthcare facilities is lower than that of other Steps. A deeper knowledge of maternal rooming-in experience has been advocated to identify the most effective rooming-in policies, thus enabling mothers to have a positive experience when practicing it in the postpartum period.

**Aim:** To investigate maternal knowledge of rooming-in and the most frequently encountered barriers and possible facilitators of adherence to the practice, according to their experience.

**Study Design and Methods:** We enrolled mothers who delivered healthy term or late preterm infants during the month of January 2019 in a tertiary referral center for neonatal care in Milan, Italy. At discharge, a structured interview about mothers' rooming-in experience was administered by healthcare professionals. Basic subjects' characteristics and mode of feeding were recorded.

**Results:** The enrolled population included 328 mothers and 333 neonates. The great majority of mothers knew of rooming-in and 48.2% practiced it continuously. The 86.3% of mothers was aware of the beneficial effects of rooming-in; promotion of mother-infant bonding, increased confidence in taking care of the baby and ability to recognize baby's feeding cues were the most frequently cited, whereas improving breastfeeding was reported by a limited number of mothers, unless they were asked a specific question about it. The main reported obstacles were fatigue (40.5%) and cesarean section related difficulties (15.5%); night was the most critical time of the day for rooming-in. Strategies suggested by mothers for improving rooming-in were increased assistance to the dyad, organizational and structural changes and the possibility to have a family member during the night. Additionally, mothers who adhered to rooming-in practice continuously during hospital stay had a higher exclusive breastfeeding rate at discharge compared to mothers who did not.

**Conclusions:** Our study contributes to a deeper knowledge of maternal rooming-in experience in an Italian tertiary maternity. We underline the importance of providing a tailored support to the mother-infant dyad in order to overcome rooming-in barriers perceived by mothers and promote a positive rooming-in experience.

## Introduction

The Baby Friendly Hospital Initiative (BFHI), launched by WHO/UNICEF in 1991 (1), is a global Initiative with the specific purpose of implementing evidence-based maternity healthcare services to protect, promote, and support breastfeeding. Since the BFHI began, its implementation has led to an improvement in the rates of initiation, exclusive breastfeeding and breastfeeding duration in many Countries (2–6).

The implementation of the BFHI strongly relies on the application of the Ten Steps for Successful Breastfeeding, first introduced in the 1989 WHO/UNICEF joint statement Protecting, Promoting, and Supporting Breastfeeding (7, 8). The evidence-based impact of the BFHI on breastfeeding (2, 3, 5, 6, 9) has shown a strong correlation with the number of Steps experienced (10), indicating that a collection of maternal practices has a stronger impact on breastfeeding than each one of them taken individually (6). Nonetheless, the degree of application of the 10 Steps varies widely between facilities (11).

Rooming-in is the practice of enabling mothers and their newborn babies to be together after birth day and night, 24 h a day, during hospital stay (8). Rooming-in has a great potential for improving mother-baby bonding, increasing mothers' confidence and decreasing psychological stress (12–14). Furthermore, sharing the room with her baby, a mother learns to recognize and promptly respond to the baby's early feeding cues, thus facilitating initiation and continuation of breastfeeding (15, 16). However, the implementation of this practice by healthcare facilities has been reported as lower than that of other Steps (such as Step 3-antenatal care, 5-breastfeeding support, and 8-responsive feeding) (11).

While organizational factors and negative healthcare professionals' attitude (11, 17) have been identified as barriers to rooming-in implementation, maternal rooming-in experience has not been extensively investigated (18) and attention has shifted from what mothers thought of rooming-in (14, 19) to what they and their babies could gain from it (20, 21). A deeper knowledge of maternal rooming-in experience has been therefore advocated to identify the most effective rooming-in policies, thus enabling mothers to have a positive experience, within a supportive environment, when practicing it in the post-partum period.

The aim of this study was to investigate the maternal knowledge of rooming-in and the most frequently encountered barriers and possible facilitators of adherence to the practice according to their experience.

## Materials and Methods

### Design and Setting

We performed a survey study during the month of January 2019 in the postnatal unit of a tertiary referral center for neonatal care (Fondazione IRCCS Ca' Granda Ospedale Maggiore Policlinico, Milan, Italy) that covers around 6,000 deliveries per year. The Fondazione promotes and supports breastfeeding in all mother-infant dyads throughout the hospital stay, following the principles of the BFHI (1). According to our internal protocol, term (37^0/7^-41^6/7^ weeks) and late preterm (34^0/7^-36^6/7^ weeks) infants with a birth weight ≥1,800 g stay in the same room as the mothers provided that their clinical conditions are stable. To facilitate the stay of mothers and their babies in the same room as continuously as possible, mothers are supported and guided through the following strategies. At admission, all mothers are informed about the importance of rooming-in and the role of the nursery as a place where they can receive help and advice, if needed. Mother-baby contact is encouraged especially at night: healthcare professionals are trained to provide indications and support to mothers to ensure they breastfeed during the night, informing them of the value, both quantity and quality-wise, of night-time feedings, and showing them how to breastfeed while lying down. Partners are allowed in the dyad's room from 8 am to 8 pm, in a family centered care perspective, whereas other family members and friends can visit from 12 pm to 8 pm (with the suggestion of limiting the number of people visiting to a maximum of 2 at a time).

Mothers and other family members are thoroughly informed on newborn safety policies, including the prevention of accidental falls and post-natal collapse. All routine care is performed in the mother-baby room, with the exception of the pre-discharge metabolic screening, which is performed at the nursery, in the presence of at least one parent. Our post-natal unit comprises 32 double rooms and a triple room, which can host a total of 67 mothers; each room has its own private bathroom. The infant's crib is placed beside the mother's bed. The average length of hospital stay, in the absence of maternal or neonatal medical complications, is 72 h after spontaneous deliveries and 96 h after cesarean sections.

The institutional Ethics Committee approved the present study and mothers provided written informed consent.

### Sample

Out of the 466 mothers who delivered healthy term and late preterm neonates during the month of January 2019, we enrolled 328 mothers with an adequate oral comprehension of the Italian language. We considered as exclusion criteria any clinical condition of the newborn requiring specific support or the need to be monitored (hospitalization in the neonatal intensive care unit, congenital malformations, genetic conditions, respiratory problems, neurologic diseases, metabolic conditions, or gastrointestinal problems) or any possible maternal condition that would pose an obstacle to rooming-in (diseases or complications requiring attendance in other Units, severe psychiatric conditions, drug abuse).

### Data Collection and Procedures

At discharge, enrolled mothers were administered a structured interview, which lasted ~10 min, by two healthcare professionals. The responses were transcribed as mothers answered, but not audio-recorded. The structured interview was previously administered to a sample of 50 Italian mothers and 10 foreign Italian-speaking mothers, whose answers were not included in the analysis, in order to identify any possible issue in the items' comprehension.

Specifically, the interview consisted of 10 items, both closed-ended and open-ended questions (Table 1). The first 3 items investigated maternal attendance to prenatal classes, their knowledge of rooming-in [as defined by United Nations Children's Fund & World Health Organization (8)] and awareness of its benefits [as described by UNICEF (22)]. Item number 4 asked to state the positive effects of this practice and item number 5 explicitly inquired if mothers were aware of the impact of rooming-in on their infant feeding practices. Items 6 and 7 investigated which time of the day mothers preferred not to practice rooming-in and for how long. Item number 8 asked if mothers had encountered any kind of difficulties during rooming-in practice, and item number 9 asked to specify which those problems were. Finally, in item number 10 mothers were encouraged to suggest strategies to improve rooming-in practice. Infants' computerized medical charts (Neocare, i & t Informatica e Tecnologia Srl, Italy) and obstetric charts were used to collect the basic characteristics of newborns and mothers (i.e., gestational age and birth weight, ethnicity, maternal age and education, parity), mode of feeding and rooming-in duration. Hospital staff keeps daily records of rooming-in time in infants' medical charts. According to the WHO definitions (23, 24) we considered exclusive breastfeeding the administration of no other food or drink, not even water, except breastmilk, and rooming-in the practice of enabling mothers and infants to be together 24 h a day during hospital say. Going to the nursery to ask for help or advice was not considered “no rooming-in.”

**Table 1:** Interview investigating maternal knowledge of rooming-in, barriers and strategies.

**WHAT MOTHERS KNOW ABOUT ROOMING-IN**
**1 Did you attend a prenatal class during this pregnancy?**
Yes
No
**2 Do you know what rooming-in means?**
Yes
No
**3 Are you aware of rooming-in benefits?**
Yes
No
**4 Can you name any positive effect of rooming-in?**
Open question
**5 Do you think that rooming-in could impact your infant feeding practices?**
Yes
No
**NO ROOMING-IN**
**6 At which time of the day were you and your baby separated?**
Morning
Afternoon
Night
Two of the above combined
All day
**7 For how long?**
1–2 h/day
3–6 h/day
> 6 h/day
**ROOMING-IN BARRIERS**
**8 Did you find it difficult to practice rooming-in?**
Yes
No
**9 Which difficulties did you encounter?**
Open question
**STRATEGIES TO IMPROVE ROOMING-IN PRACTICE**
**10 Could you suggest any strategy to improve rooming-in?**
Open question

### Statistical Analysis

Categorical variables were expressed as numbers (frequencies) and compared using the χ2 test. Continuous variables were expressed as mean ± standard deviation (SD) and tested between subgroups with the independent samples *t*-test and non-parametric tests, as appropriate. The subgroups analyzed were: Italian mothers vs. foreign mothers, primiparous vs. multiparous, mothers ≤34 vs. >34 years (mean age of the population), maternal years of education ≤13 (primary school, secondary school or high school diploma) vs. >13 years (university degree). A *p* < 0.05 was considered statistically significant.

The data reported in the open-ended questions (4, 9, 10) items were categorized by content analysis into similar ones.

Statistical analysis was performed with SPSS version 21 statistic software package (SPSS Inc., Chicago, IL, USA).

## Results

The enrolled population included 328 mothers and 333 neonates with 10 pairs of twins. Of the eligible population (466 mothers), 38 mothers declined participation to the study and 100 mothers were excluded according to the exclusion criteria. The basic characteristics of the mother-infant dyads are summarized in Table 2. The majority of the study population was Italian and had a high educational level. Over half of the mothers were primiparous and had a spontaneous delivery. With regards to the infants' population, a very limited number of newborns were late preterms.

**Table 2:** Basic characteristics of mothers and infants.

**Mothers (*****n*** **=** **328)**			
**SOCIODEMOGRAPHIC FEATURES**			
	**mean** **±** **SD (min–max)**		
**Maternal age (years)**	34.0 ± 4.9 (17-49)		
	***N*** **(%)**		
**Maternal ethnicity**			
Italian mothers	263 (80.2)		
Foreign mothers	65 (19.8)		
European	28 (8.5)		
American	20 (6.1)		
African	8 (2.4)		
Asian	9 (2.7)		
**Level of education**			
≤ 13 years	134 (40.9)		
>13 years	194 (59.1)		
**DELIVERY AND PERIPARTUM EXPERIENCES**			
**Parity**			
Primiparous	168 (51.2)		
Multiparous	160 (48.8)		
**Type of delivery**			
Spontaneous	181 (55.2)		
Vacuum/forceps	15 (4.6)		
Emergency cesarean section	47 (14.3)		
Elective cesarean section	85 (25.9)		
**Rooming-in**			
Yes	158 (48.2)		
No	170 (51.8)		
**Exclusive breastfeeding**	**Rooming-in (*****n*** **=** **158)**	**No rooming-in (*****n*** **=** **170)**	***p*****-value**
At discharge	132 (83.5)	112 (66.0)	<0.001
**Neonates (*****n*** **=** **333)**			
	**mean** **±** **SD (min–max)**		
**Gestational age (weeks)**	38.6 ± 1.6 (34-41)		
Birth weight (g)	3263 ± 518 (1965–4595)		
	***N*** **(%)**		
**Singleton**	323 (96.0)		
Late preterm	5 (1.5)		
Small for gestational age	30 (9.0)		

During hospitalization, nearly half of the mothers practiced continuous rooming-in as defined by UNICEF/WHO. Rooming-in rates were significantly higher in non-Italian mothers compared to Italian ones and in multiparous mothers compared to primiparous ones (62.5% vs. 44.0%, *p* = 0.012, and 53.5% vs. 41.6%, *p* = 0.032, respectively), whereas no difference in rooming-in rates was found according to age (48.6% vs. 46.5%, *p* = 0.73) and years of education (42.2 % vs. 45.5%, *p* = 0.61). As for mode of delivery, rooming-in rates were significantly higher in mothers who delivered vaginally than in mothers who underwent a cesarean section (56.3% vs. 34.4%, *p* < 0.001).

At discharge, the exclusive breastfeeding rate was higher in the dyads who practiced rooming-in (83.5% vs. 66%, *p* < 0.001).

The answers to the items assessed in the interview are shown in Table 3. Most mothers attended prenatal classes and were aware of rooming-in practice and its benefits. Among the reported positive effects of rooming-in, promotion of mother-infant bonding, increased confidence in taking care of the baby and ability to recognize baby's feeding cues were the most frequently cited. Improving breastfeeding was reported as a positive effect of rooming-in by a limited number of mothers, but when asked as a specific question the majority of them stated that they thought rooming-in could impact their infant's feeding practices.

**Table 3:** Answers to the interview investigating maternal knowledge of rooming-in, barriers, and strategies.

**WHAT MOTHERS KNOW ABOUT ROOMING-IN (*n* = 328)**	***N* (%)**
**1 Mothers who attended prenatal classes**	210 (64.0)
**2 Mothers aware of rooming-in practice**	297 (90.5)
**3 Mothers aware of rooming-in benefits**	283 (86.3)
**4 Positive effects mentioned (*n* = 283)**	
Encouraged mother—infant bonding	122 (43.1)
Increased confidence in taking care of the infant/Ability to recognize feeding cues	78 (27.6)
Improved breastfeeding	24 (8.5)
Reduced risk of SIDS	2 (0.7)
Two of the benefits mentioned above	47 (16.6)
Three of the benefits mentioned above	2 (0.7)
Do not remember	8 (2.8)
**5 Mothers aware of the impact of rooming-in on infant's feeding practices**	313 (95.4)
**NO ROOMING-IN**	170 (51.8)
**6 Time of day when separated**	
Morning	6 (3.5)
Afternoon	4 (2.4)
Night	133 (78.2)
Two of the above combined	8 (4.7)
All day	19 (11.2)
**7 Length of separation**	
1–2 h/day	46 (27.1)
3–6 h/day	73 (42.9)
>6 h/day	51 (30.0)
**ROOMING-IN BARRIERS**	328 (100)
**8 One or more difficulties encountered**	116 (35.4)
**9 Difficulties mentioned**	
Fatigue	47 (40.5)
Discomfort when moving after cesarean section	18 (15.5)
Pain	12 (10.3)
Perception of low milk supply	9 (7.7)
Problems with breastfeeding initiation	3 (2.6)
Two of the above combined	11 (9.5)
Three of the above combined	4 (3.4)
Other	12 (10.2)
**STRATEGIES TO IMPROVE ROOMING-IN PRACTICE**	328 (100)
**10 Strategies suggested**	
Presence of a family member during night-time	26 (7.9)
Increased assistance to mothers	64 (19.5)
Organizational and structural changes	57 (17.4)
Two of the above combined	34 (10.4)
Three of the above combined	3 (0.9)
Other	26 (7.9)
No changes needed	118 (36.0)

Nearly half of the mothers who did not practice continuous rooming-in declared that the duration of the separation was 3–6 h, while one third of them kept the baby at the nursery for more than 6 h. The majority of mothers who did not continuously room-in with their babies indicated that separation from their babies occurred during night. The occurrence of rooming-in barriers was reported by 35.4% of mothers, with fatigue after delivery being the most important difficulty encountered, followed by discomfort when moving after cesarean section and pain after delivery, respectively.

Among the strategies proposed by mothers to improve rooming-in practice, increasing support to mothers, the need for organizational and structural changes, such as the possibility to have the bathroom and the changing table inside their room, more comfortable beds, a closer nursery, the reduction of visiting hours and the presence of a family member during night were the most frequently suggested. The 10.4% of mothers indicated two or more of these strategies combined whereas 36% of mothers did not consider any changes to be necessary.

## Discussion

The present findings indicate that, although the mothers enrolled in the present study were familiar with the concept of rooming-in, they lacked a thorough understanding of its importance and associated benefits, and showed a suboptimal adherence to it in almost half of the cases. We also found that fatigue and cesarean section related difficulties were the most frequently reported barriers to rooming-in, with night being the most critical time of the day for this practice. The main strategy suggested for improving rooming-in was increasing assistance to mothers.

It is recommended that mothers' education on the basics of newborn care begins during pregnancy and continue in the immediate post-partum period (25). In our population, more than half of the mothers attended prenatal classes and almost all of them knew the UNICEF/WHO definition of rooming-in. Nevertheless, consistently with previous data published in the literature (26), less than half of them continuously practiced it. Previous studies have noted a similar discrepancy between knowledge and practice, which results in a suboptimal application of the information provided (27, 28). The time gap between the lessons attended and the birth of the baby has been suggested as one possible cause of this phenomenon (27). The results of the present study underline the lack of mothers' deep understanding of rooming-in, especially with regards to its associated benefits, which could have further negatively contributed to the suboptimal adherence to rooming-in. On the basis of the present findings, it is therefore desirable that healthcare professionals promote rooming-in to mothers highlighting its evidence-based benefits, focusing on the major role played by rooming-in in promoting breastfeeding initiation and continuation (21, 29). Consistently, a positive relationship between rooming-in and exclusive breastfeeding rates initiation was found also in our population.

When promoting rooming-in to mothers, it is important to take many aspects into consideration, both at a sociocultural and individual level. Previous studies have addressed the issue of how cultural background can influence the perception of rooming-in and the choice and modality of practicing it (30, 31), as well as impact the implementation of the BFHI itself (31). Accordingly, in our study, non-Italian mothers practiced rooming-in more than their Italian counterparts, confirming the importance of the cultural background within this context. Moreover, in the Italian subgroup we found no association between the choice to room-in and maternal age or level of education, suggesting a cross-sectional tendency not to room-in among these women.

With regards to individual factors, it is important to identify the most vulnerable women, who may need additional guidance and support in the peri-partum period. Operators should be able to acknowledge obstacles, both physical and psychological, limiting adherence to rooming-in and act in order to limit their impact. Possible physical difficulties are usually related to delivery mode and resulting maternal conditions (32, 33). Fatigue, pain due to surgery and movement difficulties, especially frequent after a cesarean section, can limit a mother's ability to take care of her newborn, thus being perceived as barriers in the practice of rooming-in. Moreover, attention should be focused also on first time mothers since primiparae have higher post-partum levels of anxiety than multiparae (34), with inexperience possibly exacerbating what already is an emotionally challenging situation for most women (35). Accordingly, in our population, first time mothers practiced rooming-in less than multiparous women. These results further underline the importance of providing additional guidance to primiparae in their transition to motherhood.

In line with the findings of the present study, mothers usually preferred to leave their infants at the nursery during the night. These results could be at least partially explained by an anticipated fear of disturbed sleep as highlighted in previous studies (36, 37). Nevertheless, it has been demonstrated that mothers who leave their babies at the nursery during the night do not have a longer or better sleep (38), while babies who room-in have a less indeterminate, more quiet sleep than those left at the nursery (39). It is therefore advisable for healthcare professionals to underline this aspect when addressing the issue of rooming-in with mothers. Increased staff attention toward the limitation of unnecessary maternal sleep interruptions due to night-time assessments and medical care has also been suggested (18).

Ultimately, mothers need more assistance, as the majority of our study population suggested. This can come either from the healthcare personnel or from the family. Staff shortage is one of the recognized barriers to the implementation of the BFHI (11, 40). A skewed healthcare professional:dyad ratio inevitably limits the time to dedicate to mothers and the problems they report. On the other hand, visits in the post-natal unit can be a double edged weapon: while partners are much needed to support and help mothers, having too many people around may affect the establishment of a new family routine.

The strength of the present study was that it addressed a relatively large number of mothers, who received the same modality of support. However, our data were collected from a single Italian tertiary center, thus these results may not be generalizable to all post-partum mothers. Moreover, we acknowledge that oral interviews could imply potential bias in comparison to written anonymous self-administered questionnaires.

## Conclusions

Our study contributes to a deeper knowledge of maternal rooming-in experience in an Italian tertiary maternity.

We underline the importance of providing a tailored support to the mother-infant dyad in order to overcome rooming-in barriers perceived by mothers and promote a positive rooming-in experience.

## Data Availability Statement

Access to the dataset generated and analyzed for this study is restricted to protect patient confidentiality and participant privacy. The data are available upon request to the corresponding author, with permission of the third party.

## Ethics Statement

The study was reviewed and approved by the Ethics Committee of the Fondazione IRCCS Ca' Granda Ospedale Maggiore Policlinico, Milan, Italy. Written informed consent to participate in this study was provided by all participants.

## Author Contributions

AC contributed to the design of the study and drafted the initial manuscript. BC and JC contributed to the interpretation of results and drafted the initial manuscript. DM contributed to the interpretation of results and reviewed and revised the manuscript. MD collected the data and contributed to the interpretation of results. MB contributed to the interpretation of results, reviewed, and revised the manuscript. DC carried out the statistical analysis and contributed to the interpretation of results. LC and LZ contributed to design the study, supervised the data collection, and reviewed the manuscript. EB, PS, FM, and LP contributed to design the study and reviewed and revised the manuscript. MG contributed to design the study, supervised data collection, contributed to the interpretation of results, reviewed and revised the manuscript. All authors approved the final manuscript as submitted.

### Conflict of Interest

The authors declare that the research was conducted in the absence of any commercial or financial relationships that could be construed as a potential conflict of interest.
